# Nurturing the next generation of pediatric physician scientists: the Padova Physician Scientist Research Training for pediatric residents

**DOI:** 10.1007/s00431-023-05258-9

**Published:** 2023-10-18

**Authors:** Alfonso Galderisi, Silvia Bressan, Liviana Da Dalt, Giorgio Perilongo, Eugenio Baraldi

**Affiliations:** 1 Pediatric Residency Program, Department of Woman and Child’s Health, University of Padova, Via N.Giustiniani, 3, 35128 Padova, Italy; 2 Department of Pediatrics, Yale University, New Haven, CT USA; 3 Institute of Pediatric Research “Città Della Speranza”, Padova, Italy

**Keywords:** Italy, Pediatrics, Physicians

## Abstract

Less than 2% of physicians complete a research training (PhD) after the residency with a declining trend in those pursuing a clinical scientist pathway in pediatrics. The exposure to research methodology during the clinical training may play a role in engaging the next generations of pediatric physician scientist. Herein, we describe the experience of the Padova Physician Scientist Research Training (PPSRT) of the pediatric residency program at the University of Padova. The PPSRT was addressed to residents attending PGY2 to PGY4 of the pediatric program and consisted of two cores: a general one including in person or virtual lectures about research methodology in pediatrics including design of a clinical trial, writing of a scientific paper and statistical methods, and a subspecialties core for the discussion of research challenges in each area and the scientific writing activities. The perceived barriers to a research training and an evaluation of the program were assessed by an anonymized questionnaire. Sixty-four out 150 residents registered for the research training with 62/64 completing the two cores. The major perceived barrier to research during clinical training was the absence of protected time (89%) followed by the lack of specific funds (37%). The group activities lead to the publication of 24 papers.

*    Conclusion*: This is the first experience in the Italian pediatric training of a dedicated research program within the frame of postgraduate medical education. Our report highlights the need for protected time to promote research interest and nurture a new generation of physician scientists.

**Table:** 

**What is Known:** *• Training to medical research is not part of residency program*. *• The declining trend of physician scientists might be reverted by early exposure to research methodology and challenges during residency*.	
**What is New:** *• An early exposure to research training during pediatric residency increases the research engagement of pediatric residents*. *• The lack of protected time for research is perceived as the major barrier to research training during residency*.

**Supplementary Information:**

The online version contains supplementary material available at 10.1007/s00431-023-05258-9.

## Background

A description of physician scientist as an endangered species was first provided in the late 1970s [1]. The term physician scientist is referred to individuals trained in both clinical medicine and in a scientific discipline bearing, in addition to their clinical duties, a role as scientist either in basic or clinical and translational research. Over the following years, the number of physician scientists has dramatically declined in the USA with only 1.5% of physicians considering research to be their primary focus [2]. In Italy, only 2.5% of medical graduates apply for a PhD program within 5 years from graduation, generally during the last year of residency or right after its completion and less than 2% complete the training [3].

Pediatrics has been particularly affected by the decline in physician scientists [4]. While several country-specific determinants play a role in this phenomenon, the time to the first research training exposure is expected to be a key determinant of the career trajectory of pediatric trainees who may potentially be interested in pursuing a research career. In Europe, pediatric clinical and research training are still largely heterogeneous [5]. Time to first applications for early career investigator European research grants is generally after the completion of the medical training (residency/fellowship) and the PhD, with medical scientist applicants for their first starting grant being older than 35 years of age [6].

The pediatric residency program of the University of Padova (Italy) has attempted, over the past 5 years, to encourage trainees to pursue a career in research by developing a clinical scientist program, which includes an early milestone during the residency program aimed to provide fundamentals of research in pediatrics to the residents and the fellows.

Herein, we describe the structure of our clinical scientist program for pediatric residents and fellows and report on the perceived barriers to engage pediatric trainees in clinical research.

## Methodology

**Fig. 1:**
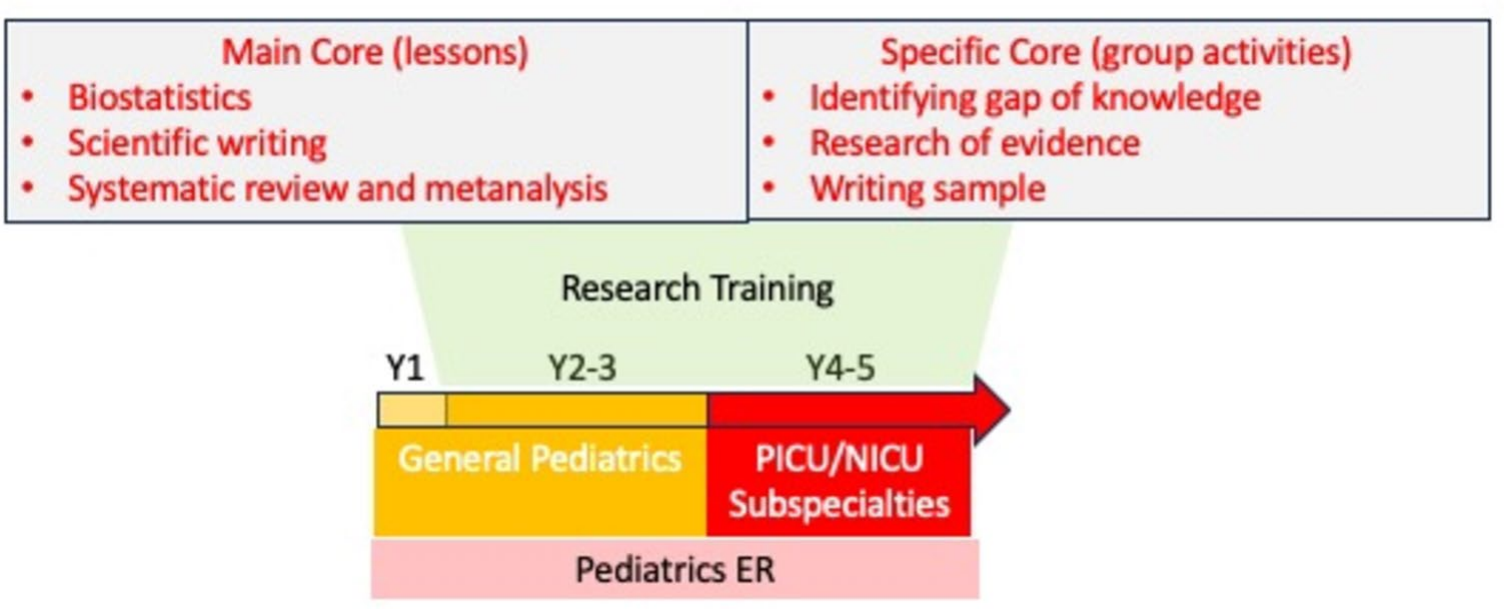
Outline of the pediatric residency program of the University of Padova

The pediatric residency of the University of Padova (Italy) is a 5-year clinical training program and includes ~ 150 residents (30/year). The program consists of a first part (2.5 years) of clinical training in general pediatrics and pediatric specialties and a second part (8–12 months) is generally dedicated to pediatric or neonatal intensive care and to an elective pediatric subspecialty (12–18 months) (Fig. 1).

The clinical scientist program targeted residents attending Y2 to Y5 of the pediatric residency program of the University of Padova (Italy).

Those who completed the clinical scientist training program filed an online anonymous questionnaire through the Moodle-Platform of the University of Padova.

At the end of the 12-month program, we explored the perceived barriers to engaging in clinical research during the training period for residents and fellows, through a multiple-choice questionnaire (Supplemental Table 1). The answers included a 1-to-4 score with 1 = not-at-all, 2 = very unlikely, 3 = likely, and 4 = very likely. For analytical purpose, a score of 1–2 was considered as a “negative” answer, while 3–4 was labeled as positive answer in a dichotomic analysis.

The estimated time to complete the survey was 12–15 min. Participants were invited to complete the questionnaire within 2 weeks from the last class of the program.

### Clinical scientist pathway at the pediatric residency program—University of Padova: program outline

The program consisted of two cores: a general common core and a group based subspecialty specific core. The general core of in person or virtual lectures targeting research methodology in pediatrics, design of a clinical trial, writing of a scientific paper with a special emphasis on systematic reviews and three statistical labs (for a total of 8 h), a lecture on systematic review methodology (in collaboration with the Cochrane program). The small-group core was aimed at providing subspecialty specific knowledge of area-specific research challenges and research methodology. Each small group consisted of 3-to-5 trainees with a faculty tutor. The groups were expected to develop an area-specific research question and to conduct a systematic review of the available evidence with the ultimate purpose of writing and publishing their work.

## Results

### Enrollment

Participants attending Y2 to Y5 were invited to register for the program on a voluntary unrestricted basis. We did not secure any protected time for the activities related to the program; however, exemption from clinical duties was guaranteed to attend the training sessions. We enrolled 64 participants out of 150 residents (43%); 62 [23 attending Y2–3 and 39 Y4–5] out of 64 completed the whole program activities including the submission of a manuscript and completed the final assessment questionnaire.

Participants who registered for the program completed a final anonymous online survey aimed at exploring the determinant of participation and the perceived barriers to attend a research training during their residency program.

### Tutoring and group composition

Participants were mostly trainees at Y4–5, while faculty included senior MD PhD (last year PhD student) and faculty (from assistant to full professors) as well as nonacademic hospitalists working at the University Hospital of Padova.

Eighty percent of participants had never had previous research or writing experience; however, more than 50% of participants declared to be interested in pursuing a research training/career after completing the clinical scientist pathway.

### Perceived experience and outputs

The program retention rate was > 90% with 62 out of 64 completing the program. More than 70% of participants perceived a moderate to large improvement of their clinical knowledge from participating in the program. The major perceived barrier to research during the clinical training program was the absence of protected time (89%) followed by the lack of specific funding (37%). The program was seen as an enhancement for the collaborative spirit within the group and as a tool to improve clinical knowledge. Only 14% of the participants felt the program was discouraging from clinical research. The group activities lead to the submission of 24 research papers with 22 accepted for publication in peer-reviewed journals within 12 months from the completion of the training. The first author of each paper was a resident and the publication’s cost was covered by the residency program.

### Perceived barriers to research during the postgraduate training

**Fig. 2:**
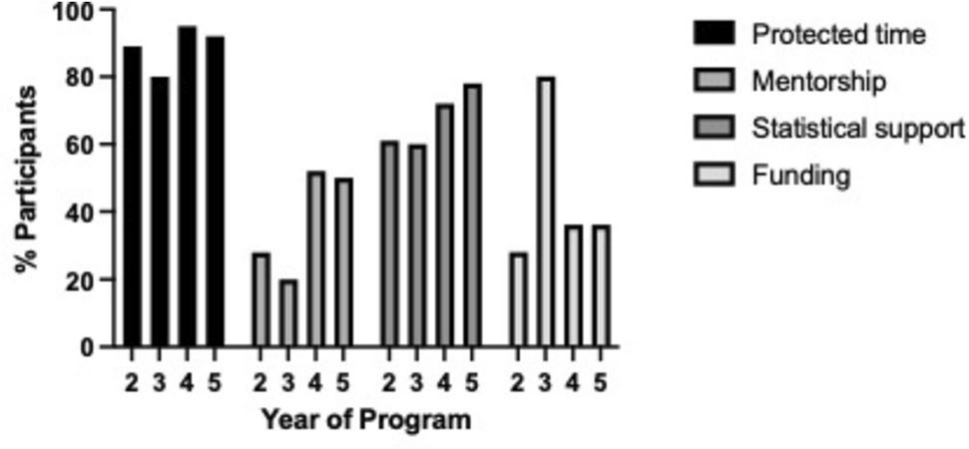
Barriers to engaging in research activities during pediatric residency/fellowship

As displayed in Fig. 2, the presence of conflictual activities during the program training was the main obstacle to participating to the program (90%) followed by the absence of dedicated statisticians for research (69%). The absence of mentorship was described as a barrier to research by the 42% of participants equally distributed across the 4 years. The lack of dedicated funding for in-training research activities was perceived as a barrier mostly by the residents of Y3 (80% vs 37% for Y4).

## Discussion and conclusion

Early exposure to research methodology training during postgraduate medical education promotes interest in pursuing a research training in residents. More than 50% of the participants to the physician scientist program of our department declared an interest in pursuing a research training, this meaning that ~ 20% of the residents may want to apply for a PhD after completion of the clinical training. This is almost 10 times higher than the average rate of applications observed in our country[3]. However, the absence of dedicated time to research activities still represents a major barrier.

An association between early research training during residency and pursuit of subspecialty fellowship training has been reported[7, 8] even though the American Academy of Pediatrics found in its 2010–2014 Annual Surveys of Graduating Residents that < 30% of respondents felt their residency programs prepared them to pursue research[9]. The need for assuring adequate clinical exposure during residency and fellowship has to be accounted while designing research-oriented programs within the frame of residency and fellowship.

Our experience represents a single-site research training and may not be generalized to other programs. However, it highlights the positive engagement that early exposure to a research training has on motivating residents and fellows to pursue a research pathway. Postgraduate training programs may want to consider the described barriers to promote research exposure of trainees and build the next generation of clinical scientists.

While the absence of fundings has not been perceived as a barrier by most of the participants, 80% of those attending Y3 listed it as an obstacle to pursue a research path. This is probably due to the specific characteristics of our program, since Y3 corresponds to the transition from general pediatrics to subspecialty trainings and the access to dedicated funding is allowed only once participants have established themselves in the research track of a fellowship.

In conclusion, we described the first experience in the Italian pediatric training of a dedicated research program within the frame of postgraduate medical education. Our report highlights the need for protected time to promote research interest and commitment in trainees attending a pediatric program.

### Supplementary Information

Below is the link to the electronic supplementary material.

Supplementary file1 (DOCX 20 KB)

## Data Availability

Data can be accessed upon request to the corresponding author.
