# Supplementary material for: Nurturing the next generation of pediatric physician scientists: the Padova Physician Scientist Research Training for pediatric residents
Source: Eur J Pediatr. 2023 Oct 18;183(4):1567–70. doi: 10.1007/s00431-023-05258-9 (PMC11001748; doi:10.1007/s00431-023-05258-9)
Supplement: Supplementary file 1 — Supplementary file1 (DOCX 20 KB) [file 431_2023_5258_MOESM1_ESM.docx]

1. **Something about you**

Age :

Gender :

Year of residency (1 to 5):

Are you enrolled in an elective program ? Y/N

Have you completed the intensive care trainining (NICU or PICU)? Y/N

Do you have previous research experience ? Y/N

Have you ever participated to the writing of a scientific article? Y/N

Have you been involved in a training program for scientific writing? Y/N

Would you be interested in a “research pathway” after residency? Y/N

1. **Perceived barriers to research in pediatric residency**

Lack of Time to participate in research training

1. Not-at-all
2. Very unlikely
3. Likely
4. Very likely

Lack of Time to conduct research study

1. Not-at-all
2. Very unlikely
3. Likely
4. Very likely

Lack of Statistical support

1. Not-at-all
2. Very unlikely
3. Likely
4. Very likely

Lack of funding to conduct research during the residency program

1. Not-at-all
2. Very unlikely
3. Likely
4. Very likely

Lack of faculty mentorship

1. Not-at-all
2. Very unlikely
3. Likely
4. Very likely

Lack of support from the Program director

1. Not-at-all
2. Very unlikely
3. Likely
4. Very likely

Lack of support from my tutors

1. Not-at-all
2. Very unlikely
3. Likely
4. Very likely
5. **The core trainings of the Physician Scientist Early Research Training**

The objectives of the program were clearly defined

1. Not-at-all
2. Very unlikely
3. Likely
4. Very likely

The time allocated to each core was adequate

1. Not-at-all
2. Very unlikely
3. Likely
4. Very likely
5. **The small-group activities (subspecialties’ core)**

The roles within the groups were well defined :

1. Not-at-all
2. Very unlikely
3. Likely
4. Very likely

The group activities were well organized

1. Not-at-all
2. Very unlikely
3. Likely
4. Very likely

My group included different skills and competences (senior and young residents, residentts with previous research experience)

1. Not-at-all
2. Very unlikely
3. Likely
4. Very likely

The time allocated to the group activities was adequate

1. Not-at-all
2. Very unlikely
3. Likely
4. Very likely

The activities did not conflict with the clinical training

1. Not-at-all
2. Very unlikely
3. Likely
4. Very likely
